# A new self-report questionnaire called "ABC" to evaluate in a clinical practice the aid perceived from services by relatives, needs and family burden of severe mental illness

**DOI:** 10.1186/1745-0179-3-15

**Published:** 2007-09-18

**Authors:** Franco Veltro, PierLuigi Morosini, Antonella Gigantesco, Massimo Casacchia, Rita Roncone, Giuseppe Dell'Acqua, Elvira Chiaia, Andrea Balbi, Renzo De Stefani, Giampiero Cesari

**Affiliations:** 1 Department of Mental Health, Campobasso, Italy; 2 National Institute of Health, Rome, Italy; 3 Institute of Psychiatry, University of L'Aquila, Italy; 4 Department of Mental Health, Trieste, Italy; 5 Department of Mental Health RM D, Roma, Italy; 6 Department of Mental Health, Trento, Italy; 7 Department of Mental Health, Arezzo, Italy

## Abstract

**Objective:**

To describe: a) a self-report questionnaire of 34 item, developed by a Family Association of Psychiatric Patients in collaboration with two psychiatrists to evaluate by key-relative in a clinical practice the perceived quality of mental health services, the needs and family burden; b) the methodology of validation.

**Methods:**

It has been studied (a) the Face Validity by two focus groups of 10 relatives for each group, (b) the concurrent validity of family burden items comparing the ABC with QPF, a widely used questionnaire, in 6 Italian mental health centres on a sample of key-relatives, (c) the discriminant validity comparing three different samples of key-relatives of patients with psychiatric illness, Alzheimer or cancer. The internal consistency of items for assessing relatives' opinions on the quality of care has been evaluated by Chronbach' s α. The test-retest has been evaluated on a sample of 20 key-relatives.

**Results:**

The results indicate a fairly good performance of the questionnaire in this preliminary but almost complete phase of validation. The time to fill in it has been estimated in a 7 minutes average.

**Conclusion:**

It is possible by this self-report questionnaire to evaluate in a clinical routine setting and in a very short time three important problems for relatives and professionals: opinions and needs of relatives, and objective and subjective family burden of severe mental illness.

## Background

All over the world mental health service providers are recognising that the involvement of consumers, caregivers and their organizations will play a meaningful role in all relevant aspects of the mental health service system, including evaluation, and research. As suggested by Chamberlin [1], services evaluation without users participation could become merely a meaningless exercise. For Chamberlin, service users involvement needs also to apply in the design of tools for evaluating care to ensure the inclusion of the aspects of psychiatric care that they deem important and the exclusion of jargon terms. Their involvement is also important because users and health professionals evaluate services in different ways. Since 1990 Brewin [2] showed that the severities of some aspects of disabilities in the community were different between staff and relatives also if were rated by the same tools. Similar results were found about needs evaluations [3,4]. According to Thornicroft & Tansella [5] "there is an emerging evidence-base that service users can make essential contributions to mental health research".

In Italy, reforms to mental health and psychiatric disability support service delivery and practice have resulted in deinstitutionalisation, recognition of fundamental human rights and changes to mental health legislation [6]. Participation by consumers and carers in service development and delivery has been viewed by governments as necessary and important in contributing to care, treatment and support systems. However, the opportunities for consumers and carer's participation in the design, development, and evaluation of services are still rare. Outcome and process evaluation of mental health services concerning very important problems for users (i.e. needs, family burden, satisfaction about intervention provided) are often promoted and conducted by research staff not by users themselves, with instruments "accurate, valid and reliable" but developed without users and carers involvement. On the other hand, research promoted and performed by users organizations, such as Eufami [7], and/or by community mental health services are very rare.

In 2006, an Italian mental health department located in Campobasso decided to use a new strategy for promoting carers participation in service evaluation. The opportunity for participation in the design, and development of an instrument for evaluating caregivers experiences and perceptions of the current mental health system was discussed with a family association called "Vivere Insieme" ("Living together"). "Vivere Insieme runs a social-health integrated service in Campobasso to fight handicaps throughout the promotion of social intervention like supported work for disabled patients and campaigns against mental illness related to stigma [8].

This instrument, which was named the 'ABC (acronym Italian of Need and Burden Assessment) for relatives', was designed to have the following features: focused on investigating domains that were considered important by relatives, with particular emphasis on their perceptions of needs; self-administered; acceptable for routine use (i.e. brief and clear).

The objective of this report is to describe the development, the main features and the validation of this instrument.

## Methods

### Preliminary version of the instrument 'ABC for relatives'

As said, the instrument has been developed by the Family Association "Vivere Insieme" of users of the Department of Mental Health of Campobasso and two of the authors (Veltro and Morosini). The members of the "Vivere Insieme" association have been trained in psychoeducational program and some of them also in the evaluation of needs. In fact they were previously involved in several investigations in this field using the Italian version of Eufami instrument, as translated and adapted by professionals and relatives of Trieste mental health department [9]. The members of the Association were asked to select the Eufami instrument items that in their opinion were the most relevant and important and to insert them in the preliminary version of the ABC instrument.

This preliminary version also included four questions covering family burden which have been suggested by the Authors because recognised as the most relevant for evaluation in this field [10-13]. Finally, items of the Rome Opinion Questionnaire (ROQ) [14] for assessing patients opinion on the quality of psychiatric care were selected by the members of the Association in collaboration with the chief of Mental Health Department of Campobasso.

### Face Validity

To assess face validity of a preliminary version, two additional focus groups consisting of 20 relatives of psychiatric patients (each group consisted of 10 relatives) in the Campobasso psychiatric service were held. Relatives were asked about their opinions on the relevance, usefulness and clarity of each item and solicited to identify which items they would eliminate if the instrument had to be shortened.

Most of these relatives commented that the items had to be expressed in a more colloquial language. They said that would prefer to have answers on a qualitative scale (e.g., from 'a little' to 'too much') instead of a temporal scale (e.g., from 'never' to 'always').

On the basis of these comments, the preliminary version was modified.

### Test-retest reliability

To assess test-retest reliability, 20 relatives of patients with severe mental disorders (except affective and organic psychotic disorders according to ICD-10) consecutively admitted to the psychiatric ward in Campobasso in the month of September 2004 were asked to fill out the ABC twice. The second completion took place 10–15 days after the first completion. Reasons for differences in the answers between the first and second completion were investigated to evaluate if something had happened that had caused a change of opinion. The instrument was presented by a social worker. Privacy was assured. Test-retest reliability was measured by calculating the intraclass correlation coefficient for each item of the instrument. Some change were made after the reliability study to develop the final version.

### Concurrent validity and internal consistency

#### a. Subjects and procedure

The study took place in 6 community mental health centres located in the North (Trieste e Trento), Centre (Arezzo, L'Aquila and Rome) and Southern Italy (Campobasso), over a period of 10-days, in June of 2005. These centres were chosen for the following reasons. The mental health departments of Arezzo and Trieste have been the first departments in Italy that have developed community programs to support relatives of patients. Trento has a good experience in self-help groups and patients' empowerment promotion. The service of Rome implemented the routine evaluation of the satisfaction with services of patients and relatives. Finally, mental health professionals of L'Aquila and Campobasso services have been active for years in psychoeducational interventions for relatives [15,16] and have also collaboration programs with relatives associations [8]. Moreover, most of the professionals working in these centres have a long experience in the evaluative health system research. The different services were invited by the mental health professionals of Campobasso to take part in the study, and all centres agreed. For each centre, over a period of ten days in the month of June 2005, afferent psychotic patients to the outpatient unit, with exclusion of organic and bipolar disorders, who were heavy users (operational definition: with at least on average one contact with service per month in the last year) were asked the permission to contact their relatives in order to administrate the questionnaire. Privacy was promised. Clinical or socio-demographic characteristics of patients do not have been collected also because not considered useful for the aim of the study. Finally, the final version of ABC were given to key-relatives and one professional was available in order to clarify the aim of the study.

#### b. Concurrent validity

Key-relatives were also asked to complete the FPQ [11]. The comparison concerned the 2 ABC items on key-relatives Objective Burden and on key-relatives Subjective Burden (see table 3, items 1 and 2). As criterion measures, the averages of the 7 items of the objective burden and of the 7 items of the subjective burden of FPQ were used. The Spearman correlation coefficient was calculated. Given that the constructs covered by the criterion measures and the 2 ABC items were overlapping but not identical, a correlation in the moderate range was expected.

#### c. Internal consistency

It has been determined by calculating Cronbach's alpha on the items concerning opinion about care quality. All the other item concerned individual constructs or have dichotomous scales.

### Discriminant validity

Other comparative studies showed different levels of family burden between relatives of patients suffering from psychosis and relatives of patients suffering from cancer or Alzheimer's illness [12]. To assess the ability of the instrument to assess different levels of burden in these different groups of relatives, it was chosen to introduce them in a service for Alzheimer disease, and in a outpatient service for cancer. We expect, according to the results of the literature, that the instrument pointed out higher levels of family burden in relatives of patients with Alzheimer compared with those observed in relatives of patients with cancer or psychosis.

Before implementation, the instrument was discussed by relevant health care professionals (3 domiciliary nurses for Alzheimer disease, and 1 social worker and 1 psycho-oncologist for cancer). These professionals suggested some modifications for the list of needs. Three out of 4 items on relatives burden were not modified.

The relatives of patients consecutively seen in a domiciliary service for Alzheimer disease, and in a outpatient service for cancer, in the two towns of Termoli and Benevento were given the questionnaire, over a period of 15 days of 2005. In that period also other relatives of psychotic and bipolar patients in Campobasso were asked to fill the original ABC. As in the concurrent validity study, and for the same reasons, data on social-demographic characteristics of patients were not collected.

Given the differences between the original ABC and the modified ABCs for somatic patients, the comparison was limited to two items about care quality, and three items on family burden.

The three groups of relatives were tested for differences by means Anova or Kruskal-Wallis.

For some comparisons, they were expected substantial between-group differences (e.g., for the item on the quality of information received about patient's disorder or illness).

All statistical analyses were run under SPSS, version 12.0 for Windows.

## Results

### Final version of the questionnaire

The final version of the instrument included 34 items. They are related to different domains: a) opinion on care quality: 8 items (table 1) with a Likert-type scale with six levels; b1) relatives' received needs of more information about the disorder or illness: 6 items (table 2), with a dicotomic scale (yes/no); b2) relatives perceptions of needs: 9 items, as answer scale a dicotomic scale was used; b3) relatives' personal needs: 7 items, with a dicotomic scale; c) relatives' burden: 4 items (table 3) on objective and family burden (1 item), key relative subjective burden (1 item), others relatives subjective burden (1 item), and expectation for the future (1 item) (answers on a 6-point Likert scale). For all the items the period of reference are the last 30 days.

**Table 1:** Test-retest reliability (n = 20) of the 8 items for assessing relatives' opinions on the quality of care (ICC = Intraclass Correlation Coefficient; CI 95% = 95% Confidence Interval)

Item	ICC	95%	CI	p <
Overall, how much are you satisfied with the treatment that P has received in this unit?	.76	.49	.89	.001
How much do you think that P is satisfied with the treatment that he/she has received in this unit?	.76	.49	.89	.001
Has the staff of this mental health unit been usually kind?	.55	.15	.79	.005
Has the staff of this mental health unit been usually available and ready to assist?	.70	.40	.87	.001
Has the staff of this mental health unit given you clear and complete information about P's disorder and treatment?	.80	.66	.94	.001
Do you think that in this mental health unit your opinion is taken into account?	.83	.62	.93	.001
Overall, do you think that P has improved since he/she came here?	.42	-.02	.72	.03
Overall, would you recommend this mental health unit to somebody with the same problems and the same history as P?	.75	.47	.89	.001

For all items the answer scale has six levels:  not at all;  little;  somewhat;  fairly;  very (or much) with some exceptions;  very (or much) without exceptions

**Table 2:** Test-retest reliability (n = 20) of the 22 items concerning relatives' perceived needs (ICC = Intraclass Correlation Coefficient; CI 95% = 95% Confidence Interval)

Item	ICC	95%	CI	P <
1) About which of the following topics would you like to have more information or instructions (*please tick no more than three options*):				
1.1) drugs and their side effects	.55	.17	.79	.001
1.2) psychotherapy and/or rehabilitation	.90	.78	.96	.001
1.3) how we relatives may help P better	.53	.13	.80	.005
1.4) P's and we relatives' rights and entitlements	.59	.23	.81	.001
1.5) P's disorder causes	.36	-.04	.67	.004
1.6) P's disorder evolution in the future	.67	.33	.85	.001

2) Which of the following treatments or changes do you think P particularly needs (*please tick no more than three options*):				
2.1) change of mental health unit, being taken care elsewhere	.90	.78	.96	.001
2.2) hospitalisation or remaining in hospital	.90	.78	.96	.001
2.3) mental health staff visiting at home	.70	.37	.86	.003
2.4) help to take drugs as prescribed	.62	.25	.83	.002
2.5) attending a day centre for rehabilitation	.50	.07	.76	.013
2.6) help in finding or keeping a job (e.g. through some vocational training)	.80	.57	.91	.001
2.7) meeting and seeing more people	.61	.25	.82	.001
2.8) going on holiday	.33	-.13	.67	.07
2.9) taking part to a patients' self-help group	.50	.07	.76	.012

3) Do you think that you or some other P's relative need particularly some of the following, because of P's situation (*please tick yes to no more than three options*):				
3.1) taking part to a relatives' association or self-help group	.50	.08	.76	.011
3.2) having more time to spend for your personal problem	.54	.15	.79	.005
3.3) having P living elsewhere for a period	-.07	-.52	.38	.62
3.4) going on holiday	.69	.38	.86	.001
3.5) receiving some help for housekeeping tasks	.33	-.13	.67	.070
3.6) receiving some help for financial problem	.47	.07	.74	.012
3.7) more collaboration with family members	.74	.45	.89	.001

For each item the answer levels are just two:  yes;  no. For each area others needs also may be reported.

**Table 3:** Test-retest reliability (n = 20) of the 4 items concerning Family Burden (ICC = Intraclass Correlation Coefficient; CI 95% = 95% Confidence Interval)

Item	ICC	95%	CI	p <
1. Overall, in the last four weeks, which practical problems has the family had as a consequence of P's condition (e.g. limitations in amount or quality of work, need to work more to cover expenses, limitations in holidays or week-ends, difficulty in pursuing one's hobbies or interests, being compelled to neglect other family members, difficulty in receiving visits from friends at home)?	. 73	.45	.88	.001
2. In the last four weeks, how often have you felt upset (e.g. depressed or anxious or very tense, nervous or sleeping badly) as a consequence of P's conditions?	.77	.51	.90	.001
3. In the last four weeks how often some other family member has felt upset (e.g. depressed or anxious or very tense, nervous, sleeping badly) as a consequence of P's condition?	.84	.62	.24	.001
4. Do you think that your and P's situations may improve (do you have hope for the future?)	.87	.71	.94	.001

The first question has the following answer scale:  none or almost none;  mild;  manifest for everyone, not yet distressing;  distressing, not yet severe;  severe, not continuous;  severe and continuous.

The second and third questions have the following answer scale:  never;  rarely;  sometimes;  more or less half of the time;  most of the time;  always

The fourth question has the following answer scale:  not at all;  little;  moderately;  = fairly;  much;  very much

### Test-retest reliability

The agreement between the first and the second completion was very satisfactory for the items concerning the opinion on care quality and on family burden. The ICC-values of the individual items for assessing relatives' perceived needs ranged from 0.33 to 0.90. If answers for which was reported that something had happened between the first and the second completion were excluded, all ICC values become higher than 0.60.

Time of completion was contained, ranging on average from about 7 minutes (first completion) to about 5 minutes (second completion).

### Concurrent validity and internal consistency

#### a. Subjects

A total of 132 key-relatives filled-out the instrument. Of these, 52% were females; the mean age was 57 ± 12 years (range: 24–83 years); 12% had 5 years of education, 30% had 8–12 years, and 42% had 13 years or more of education. Sixteen percent had a degree. Sixty-five percent were parents, 12% partners, 9% sisters, 7% sons and 7% other kind of relatives.

#### b. Concurrent validity

The correlations between the ABC items studied and their criterion measures were moderate in size. The r' Spearman correlation coefficients for objective and subjective key-relatives burden were .53 (p < .0001) and .54 (p < .0001), respectively.

#### c. Internal consistency

The Cronbach's alpha for the 8 items on the opinion about care quality was 0.90.

### Discriminant validity

The ABC has been filled out by 108 key-relatives, of which 42 (39%) were relatives of psychiatric patients, 36 (33%) of patients with cancer and 30 (28%) of patients with Alzheimer. In the three groups, the mean age were respectively 61.2 ± 9.1, 64.0 ± 12.7, 70.6 ± 9.4, and the female proportions were 57%, 72% and 77%. The relatives were partners (5%, 33% and 63%); parents (74%, 42% and 0%); sons (7%, 8% and 37%); brothers/sisters (14%, 17% and 0%).

Significant differences between groups were found with regard to opinions about treatment (Kruskal-Wallis: χ^2 ^= 16.2; df = 2; p < .0001) and opinions about received information (Kruskal-Wallis: χ^2 ^= 7.7; df = 2; p < .02). The highest levels of satisfaction were found among psychiatric patients' relatives, and the lowest among Alzheimer patients' relatives.

In the three groups the mean rank for objective burden were 40.6 (relatives of psychiatric patients'), 59.8 (relatives of patients with cancer) and 66.0 (relatives of patients with Alzheimer' illness) (Kruskal-Wallis: χ^2 ^= 17.0; df = 2; p < .0001) and for subjective burden was 49.8, 53.0 e 61.0. (Kruskal-Wallis: χ^2 ^= 2.9; df = 2; p = .23). No difference was observed between the groups about the subjective other than key-relatives burden.

## Discussion

Both reliability and validity have been assessed. The face validity has been examined by two focus groups. Concurrent and discriminant validity were investigated in a fairly large multicenter sample. Subcategories of non affective psychiatric disorders were not considered, however it is accepted that both family burden and relatives perceived needs are associated with specific problem behaviours regardless of the psychiatric diagnosis [10,17]. We believe that the main positive aspect was that the instrument was developed in close collaboration with relatives. The final version may be considered almost a self-developed instrument by relatives themselves.

The degree of test-retest reliability was satisfactory, especially for the items on quality of care opinion and on family burden. The Cronbach's alpha was 0.90, which is very satisfactory considering the limited number of items.

The concurrent validity study showed that the pattern of correlations between the ABC items on family burden and the criterion measures was consistent. As anticipated, an higher correlation could not be expected.

The discriminant analyses succeeded in differentiating the family burden and the opinion about care items between the three categories of illnesses (psychosis, cancer, Alzheimer). As observed in other studies the family burden of relatives of patients with Alzheimer was higher [12].

Unfortunately, because of unavoidable difference between the psychiatric, the cancer and the Alzheimer instrument, the items on perceived needs and most items on opinions on care quality could not be compared. No attempts were made to collect socio-demographic characteristics of patients. However, because of procedure of questionnaire administration to the relatives, it is unlikely that they were relatives of patients not representative of population in treatment in the outpatient mental health centres.

This instrument is in our opinion a practical answer to the realistic Chamberlin worries [1] ("the evaluation without a user participation could be a meaningless exercise") and to the Ohaeri suggestions [18] for the future studies ("articulating simple tools for caregiver assessment in the clinical setting").
